# A nurse-led parental self-efficacy promotion program in parents of children with autism spectrum disorder: a quasi-experimental protocol

**DOI:** 10.3389/fpsyt.2025.1411518

**Published:** 2025-09-19

**Authors:** Yushen Dai, E. Zou, Yan Ji, Xiuxian Chen, Miaoying Chen, Kaiyun Chen, Baoqin Huang, Min Xu, Qiuling Guo, Meiling Cai, Tao Deng, Yao Wei, Shaofei Liu, Lifeng Zhang

**Affiliations:** ^1^ School of Nursing, Sun Yat-sen University, Guangzhou, China; ^2^ Child Development and Behavior Center, The Third Affiliated Hospital, Sun Yat-sen University, Guangzhou, China

**Keywords:** autism spectrum disorders, self-efficacy, parents, nurses, health education, child

## Abstract

**Purpose:**

Parents of children with autism spectrum disorder (ASD) often lack the knowledge and confidence to implement early intervention. This study will evaluate the effects of a parental self-efficacy promotion program on parenting self-efficacy, parenting stress, intervention compliance, and family quality of life for parents of children with ASD.

**Design and methods:**

This two-arm, pretest, post-test quasi-experimental study recruits 80 parents in one tertiary hospital in China. The control group receives routine care, and the intervention group receives a 1-month self-efficacy promotion program on the basis of usual care. This program consists of one face-to-face session and three online sessions, supplemented by a written pamphlet. Content includes goal setting, experience sharing, verbal encouragement, and mobilizing of positive emotions.

**Conclusion:**

This study will empower the nurses as leaders to promote the well-being of parents of children with ASD and improve the early family intervention for their child by providing a parental self-efficacy promotion program.

**Practice implications:**

Parents need support to implement intervention in the family for their child with ASD. A nurse-led program targeted at improving parental self-efficacy is worth considering.

## Introduction

Autism spectrum disorder (ASD) encompasses a set of neurodevelopmental conditions with core symptoms of social interaction difficulties and stereotypical behavior (1). The prevalence of ASD has been increasing globally, with 2.8% in the United States and approximately 0.7% in China (2, 3). Early and continuous intervention is crucial for language development, social interaction, cognition, and reduction of stereotyped behaviors in children with ASD (4, 5). A study found that around 20% of individuals with ASD did not receive any intervention after diagnosis (6). Additionally, a European study revealed that 61.61% of children with ASD received intervention within 2 months of diagnosis (7), however, in southern China, less than one-third of families received home-based intervention within 1 month after diagnosis (8, 9), highlighting the need to improve early and long-term intervention rates. Parents play a vital role as primary caregivers and their fidelity in implementing early intervention significantly impacts children’s outcomes (10). Unfortunately, parents of children with ASD generally lack knowledge in implementing ASD training and exhibit lower levels of parenting self-efficacy, especially during the early stage of diagnosis (11, 12), which is positively correlated with negative coping strategies and may potentially influence the child’s progress (13). The financial burden of ASD interventions including significant costs over the lifetime of an individual, poses a considerable challenge for families and may affect access to ASD-related services and support (9, 14). Therefore, improving parents’ confidence, involvement, and intervention skills is essential to ensure the quality of early family intervention for children with ASD.

Parental self-efficacy (PSE) refers to parents’ beliefs in their ability to influence their child’s development through their process, motivation, and cognitive abilities (15). PSE directly influences parental behavior motivation and is formed and influenced by mastery experiences, vicarious experiences, verbal persuasions, and physical and affective states (16). Parents of children with ASD often experience lower levels of parenting self-efficacy compared to parents of typically developing children due to the specific symptoms associated with ASD (11). PSE is a significant predictor of family intervention for parents of children with ASD (17). Higher PSE is associated with the use of promotive parenting strategies, while lower PSE is correlated with higher levels of children’s emotional/behavioral behaviors and caregiver distress (18, 19). Moreover, lower PSE can also lead to a decrease in family quality of life (20).

The family plays a crucial role in the intervention; however, implementation of long-term early intervention is poor in most families (9). Studies have shown that PSE is positively correlated with active parenting practices (21), and more support for families to enhance self-efficacy is expected. Several studies have reported the effects of health education for parents of children with ASD (22–24), although most previous health education programs focused primarily on delivering ASD-related intervention knowledge and skills rather than targeting parental psychological mechanisms (10, 25). Ginn et al. (23) provided interactive training to 30 parent-child dyads with ASD, teaching mothers techniques to engage in social and playful behaviors with their child. This study reported significant improvements in children’s disruptive behaviors and mothers’ stress after the intervention and follow-up. Zhou et al. (26) implemented a pilot study including four times daily ASD-related rehabilitation training and psychoeducation for families of children with ASD. The results showed an improvement in parenting self-efficacy, and a decrease in anxiety and depression for parents.

Emerging studies have demonstrated that health education based on self-efficacy theory has been effective in increasing parental self-efficacy and improving disease management outcomes for parents of children with chronic diseases (27–29). For example, Cheng et al. (27) designed a randomized controlled trial (RCT) based on the self-efficacy theory for parents in controlling child’s eczema, which showed significant improvement in the child’s eczema severity and parental self-efficacy. Similarly, another RCT was conducted for parents of children with asthma, the intervention groups had significantly higher parental self-efficacy and 1.4 times the child’s experience of an asthma crisis improved compared to the control group post-intervention (da Rocha Mendes et al., 2024). However, non-significant results were also observed, an online platform was developed to enhance parental self-efficacy and of parents with a child with type 1 diabetes, the self-empowerment was not significant change post-intervention (30).

In summary, most existing ASD parents program emphasize knowledge or skills delivery (10), but relatively few have been designed primarily to enhance parental psychological such as PSE, or to target Bandura’s four sources of self-efficacy in a structured way (3). Therefore, this intervention will address the gap by testing whether a theory-driven, nurse-delivered PSE intervention improves parental self-efficacy, parenting stress, family quality of life and symptom severity post intervention and at 6-month follow-up. The hypotheses are as follows:

1. Compared with the control group, the intervention group will be expected to demonstrate higher parental self-efficacy scores at post-intervention and at 6-month follow-up.
2. Compared with the control group, the intervention group will show higher improvements in secondary outcomes at post-intervention and at 6-month follow-up, including lower parenting stress, higher treatment compliance, family quality of life, and reduced child symptom severity.

### Theoretical framework

The intervention is based on Bandura’s self-efficacy theory, which suggests that human behavior is influenced by self-efficacy expectations and outcome expectations. Self-efficacy was reflected in this study as parents’ expectation of themselves to implement family interventions as behaviors (31). There are four aspects of Bandura’s self-efficacy theory, namely mastery experiences, which comes from the individual’s personal experience and is the primary source of self-efficacy; vicarious experiences, where experienced people share their experiences; verbal persuasions, which refers to the use of persuasive language to encourage an individual and increase their self-efficacy; and physical and affective states, which are primarily physiological and emotional reactions to a task faced by individuals (31). The content includes helping parents set training goals, providing them with the necessary knowledge and skills (mastery experiences). Experienced parents will participate in group sessions to share their experiences with others, fostering a sense of learning from peers (vicarious experiences). We will employ verbal persuasion throughout the intervention to commend parents who demonstrate good compliance and support those facing challenges in identifying difficulties and exploring potential solutions. Additionally, we will address the physical and affective states of parents by providing strategies to help them manage emotional distress (16).

## Methods

### Study design

This study protocol outlines a single-center, pre-test, post-test, double-arm quasi-experimental trial with a 1:1 ratio. Randomized controlled trial was deemed infeasible in this setting because concurrent enrollment to parallel arms would likely result in contamination in shared ward and the same nurse educators. The Standard Protocol Items: Recommendations for Interventional Trials can be found in Supplementary File 1. The control group received routine care, and the intervention group received a 1-month self-efficacy promotion program on the basis of usual care. **Table 1** summarizes the program of enrollment, intervention and evaluation. The study has been registered at the Chinese Clinical Trial Registry (23/05/2023, No. ChiCTR2300071721).

**Table 1:** Protocol schedule of enrollment, interventions, and assessments.

Time point	Pre-intervention screening/consent	Allocation	Baseline measurement	PSE promotion intervention	Post-intervention Measurement	Follow-up measurement
	-T1	0	T0	Intervention	T1	T2
Eligibility screen	×					
Informed consent	×					
Allocation		×				
Intervention group			×	×	×	×
Control group			×		×	×
Descriptive characteristics						
Sociodemographic characteristics			×			
ASD-related characteristics			×			
PSOC			×		×	×
PSI-SF			×		×	×
TCS			×		×	×
FQoL			×		×	×
ABC			×			×

ABC, Autism Behavior Checklist; FQoL, Beach Center Family Quality of Life Scale; PSE, Parenting self-efficacy; PSI-SF, Parenting Stress Index-Short Form; PSOC, Parenting Sense of Competence Scale; TCS, Treatment Compliance Scale for Children with Autism Spectrum Disorder.

### Study setting and population

The study is conducted at the child development behavior center of a tertiary hospital in southern China. This center provides ASD diagnosis and structured interventions. Hospitalization is used to provide diagnostic assessment for children with ASD and initiate parent-mediated intervention for children with ASD. The typical hospitalization period is 7 to 14 days. The target population consists of parents of children who have recently been diagnosed with ASD. The study conducted from October 2022 to May 2024. Participants recruited from October 2022 to March 2023 were assigned to the control group, while participants recruited from April to October 2023 were assigned to the intervention group.

### Eligibility criteria

Parents and children who meet the following criteria will be included in the study. For parents, the inclusion criteria are (1) age ≥ 18 years; (2) living with the child; (3) educational level of elementary school or above; (4) proficiency in the use of mobile phones, WeChat, and Tencent meetings and (5) signed informed consent form. The exclusion criteria are (1) diagnosed with severe psychiatric disease, end-stage disease, or any other serious illness, such as cancer; (2) had experienced a serious traumatic event (e.g., car accident, surgery) since the ASD diagnosis; (3) participating in the same type of research; and (4) do not plan to implement the family intervention. For children, the inclusion criteria are (1) diagnosed with ASD by a certified doctor within one year, according to the DSM-5 criteria (32), (2) ASD is the primary diagnosis and (3) age ≤14 years. The exclusion criteria are (1) combined with other intellectual developmental disorders, such as trisomy 21; (2) diagnosed with severe chronic diseases, such as congenital heart disease, and receiving long-term treatment. Only one parent in each family will be included in the study.

### Withdrawal criteria

Parents will be considered withdrawn from the study if they refuse to participate or fail to undergo post-intervention evaluation. Reasons for participant withdrawals and discontinuation within the trial will be documented.

### Interventions

#### Preparation phase

**Figure 1:**
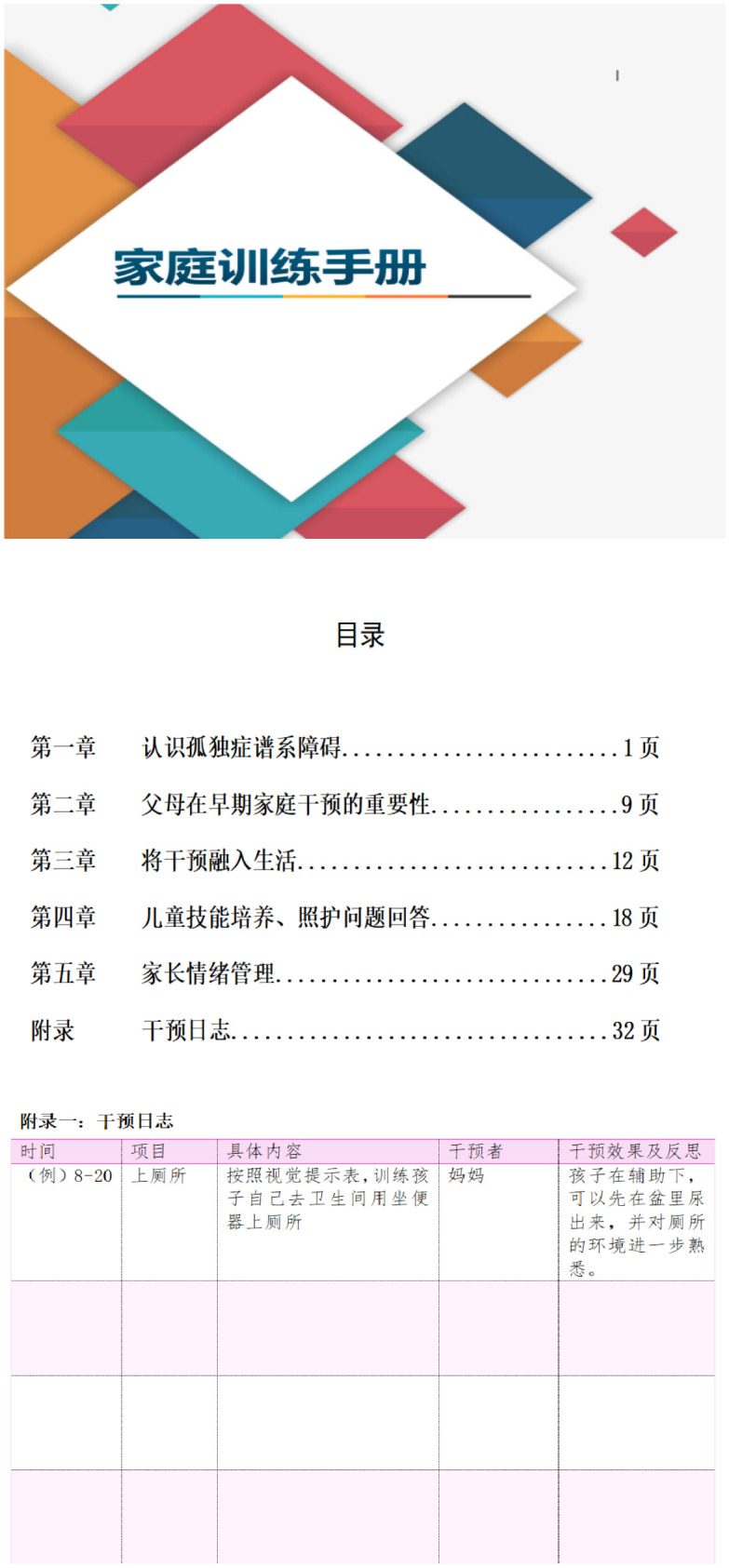
Screening shot of pamphlet.

- A nurse-led intervention team was established, consisting of nursing specialists specialized in pediatric nursing, nursing postgraduate students in pediatric nursing orientation, pediatricians, rehabilitation therapists, and senior clinical nurses experienced in ASD intervention. The nursing postgraduate students and specialists developed an initial intervention protocol, which was based on the results of the literature review and Bandura’s self-efficacy theory. Then, the second version of the protocol was validated by one pediatrician and three senior clinical nurses with extensive experience in ASD intervention.
- The pamphlet for “Family Training for Children with ASD” was formulated by the intervention team members. The pamphlet covers five aspects: understanding ASD, the importance of parents in early family intervention, integrating intervention into daily life, daily management of the child, and parental emotional management. It also includes a daily intervention record sheet for parents to document their intervention activities and note any challenges. Contact information for the department and nurses is provided in the pamphlet. In **Figure 1**, the screenshots of the pamphlet are shown.
- To facilitate communication and minimize follow-up loss, WeChat (Tencent Corp) accounts and separate WeChat groups will be created for both the experimental and control groups. WeChat, the most popular communication software in China, was chosen for its convenience and accessibility. These groups will be managed by nursing graduate students, providing a convenient way to contact parents. The WeChat groups will also be utilized to send session links (for the experimental group) and collect information from parents during the follow-up period (for both groups).

#### Control group

Parents in the control group will receive routine care, including nursing care such as toilet instruction, feeding instruction and sleep instruction provided during hospitalization. Nurses will follow up with parents through telephone calls one, two, or three months after discharge.

#### Intervention group

Parents in the intervention group will receive the parenting self-efficacy (PSE) program and routine care. A pretest involving four parents was implemented prior to the start of the intervention. The final protocol includes a total of four sessions: one face-to-face session before discharge and three online sessions at weeks 1, 2, and 3 post-discharge. Each session will last 40–60 minutes. Intervention delivery will follow a hybrid method: an in-person session during hospitalization for *in-situ* coaching, followed by three online sessions via Tencent Meeting after discharge to maintain continuity and reduce travel burden.

The first session will take place in the hospital’s meeting room before participants are discharged. The nurse will introduce herself, describe the intervention process, invite parents to join the intervention group’s WeChat group and provide a pamphlet. A PowerPoint presentation on “understanding ASD” and “the importance of parents in early family intervention” will be delivered. Subsequently, the nurse and a rehabilitation therapist will collaborate with parents to set individualized goals and content for home training based on their child’s specific needs. Lastly, parents will be instructed to complete a training record sheet to document daily training activities and reflections. The remaining three online sessions will be conducted via Tencent meetings. The second session will focus on strategies for integrating intervention into daily life and family management. The third session will focus on emotion management in the care giving process of children with ASD. The nurse and rehabilitation therapist will discuss the child’s intervention process, address challenges faced by parents in implementing and adhering to the intervention, assess achievements, and provide support in problem-solving and enhancing confidence in the intervention. In the fourth session, the nurse and parents will review the initial goals set by parents, assess progress and challenges encountered in adhering to the intervention, and discuss new goals or encourage persistence in implementing efforts. Within the group, 1–2 parents who have a proactive attitude toward the intervention and are willing to share their caring experiences may be invited to do so. They will provide insights and tips based on their experiences to facilitate learning and discussion among parents. With parental consent, a video demonstrating the parents implementing intervention for a child with ASD may be shown to inspire learning and discussion among parents. These four aspects are incorporated into each component of the intervention, as shown in **Table 2**.

**Table 2:** Parental self-efficacy promotion components, strategies, and techniques.

Components	Strategies	Content
Mastery experience	Setting achievable goals Providing information on ASD Gaining experience	(1) Based on the previous assessment (PEP-3) and behavior observation, the intervention team will work with parents to develop intervention goals and identify achievable goals for their child at home training. (2) Teaching the knowledge about ASD and family training. (3) Emphasize the importance of family intervention, and encourage parents to record the daily training content.
Vicarious experience	Experience sharing from other parents	(1) Inviting some parents to share their experience of caring for children. (2) Watching 1–2 videos of parents training their children at home. Encourage parents to learn and discuss with each other.
Verbal persuasions	Verbal encouragement, explanation, and persuasion throughout the process	(1) Give timely recognition to parents who persist in intervention to negative emotions, and encourage them to continue to do so. (2) When parents do not adhere to the intervention and encounter difficulties, help them to find out the reasons, propose countermeasures, and encourage them to adhere to home training.
Physical and affective states	Mobilizing positive emotions	(1) Mental health education: parents are instructed to manage their emotions during the caring process of children, and guide parents to seek help from psychologists if necessary. (2) Encourage parents to talk to their friends and relatives when they encounter difficulties. And encourage parents to pay more attention to their physical situations. (3) Play a 5-minute medication video about relaxation and breathing.
	Providing social support	(1) Mobilize family members to learn about the intervention and encourage family members to participate in the intervention, and create a friendly family atmosphere. (2) Provide relevant resources and introduce domestic support policies, and information on intervention services and institutions. (3) Encourage parents to promptly reflect on any questions in the WeChat group during the follow-up phase to help them solve questions.

### Data collection

**Figure 2:**
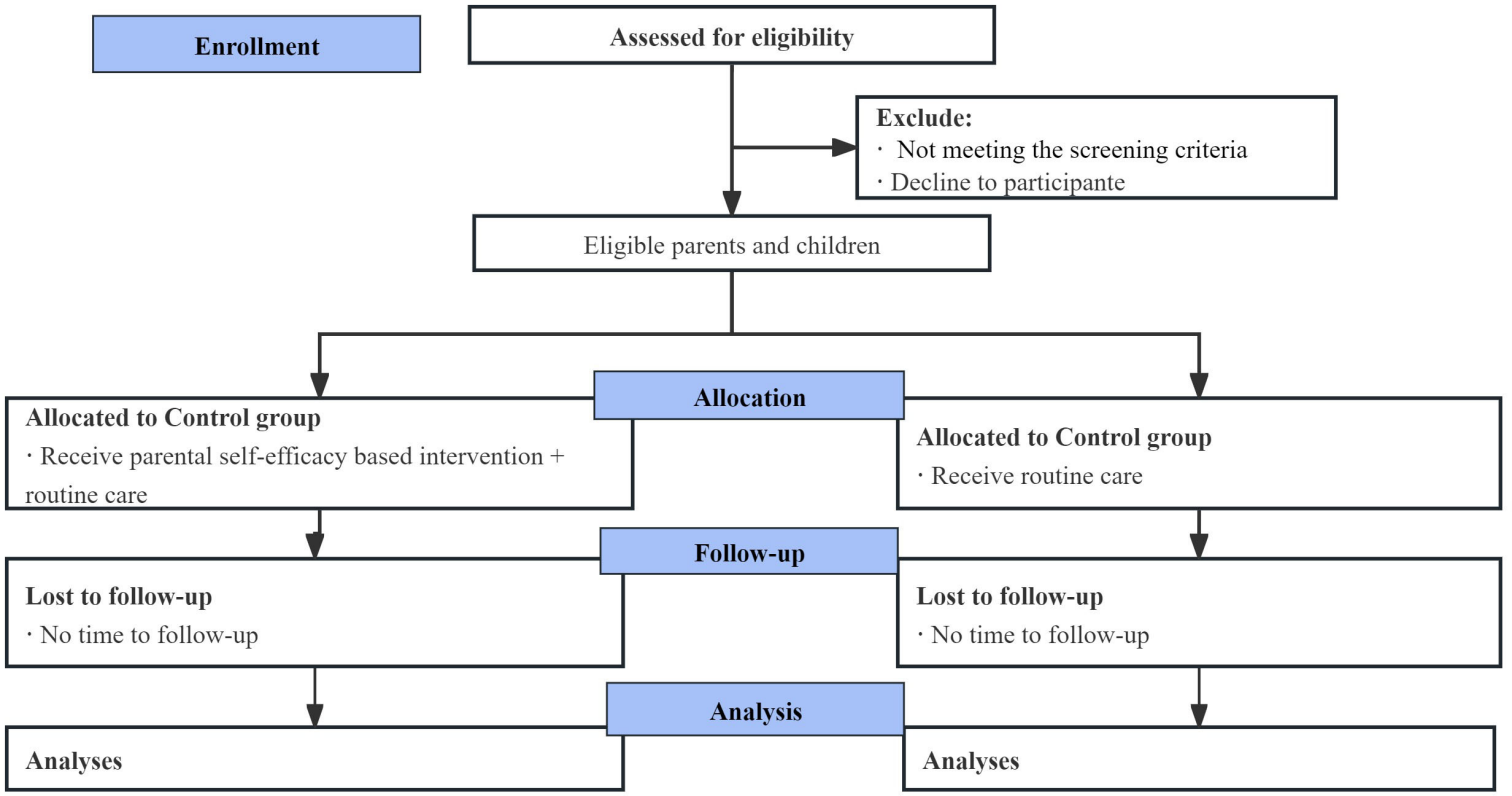
Flow diagram of the trial procedures.

The researcher will recruit parents by posting posters during the first two days of hospitalization. Eligible parents will undergo a thorough explanation of the study, and written consent will be obtained (-T1) by researchers. Two nurses trained in using standardized instructions will collect the data. Baseline data (T0) will be collected face-to-face using a paper version of the questionnaire. Post-intervention data will be collected within three days after intervention (T1) and 6 months after the intervention (T2) through the online questionnaire platform, *Questionnaire Star* (Changsha Ranxing Information Technology Co., Ltd). This platform was chosen for its ability to ensure data quality, provide an opt-out option, and allow parents to complete the surveys at their convenience. The flow diagram of the trial procedures is presented in **Figure 2**.

### Data management

To protect the confidentiality of participants, all data will be de-identified using participant codes before analysis. De-identified data will be stored on a password-protected hard disk (encrypted external drive). Access is role-based and restricted to authorized team members (PI, data manager, statistician). Data will be retained for 10 years after publication, then securely destroyed. In line with registry requirements, de-identified data will be uploaded to the ResMan (http://www.medresman.org) within six months of publication. Additional access will be available from the corresponding author upon reasonable request.

### Outcomes

#### Sociodemographic characteristics

The parents’ and their children’s sociodemographic characteristics will be collected by a self-designed questionnaire. It includes the parents’ age, sex, marital status, education level, employment status, per capita monthly income, the number of children in the family, living status, and the children’s age, sex, and education status.

#### ASD-related characteristics

Participants will be asked about the ASD-related characteristics of their child, including the child’s age at the first observed ASD-related symptoms, initial ASD-related symptoms observed by the parents, comorbid conditions, the age at ASD diagnosis, presence or absence of ASD-related training, monthly expenditure for training and payments for training expenses, presence or absence of family training, the child’s age at first presence of family training, the frequency of family training, clinic outpatient visits or not, self-reported major factors affecting the presence of intervention.

#### Primary outcome

Parental self-efficacy will be measured using the Chinese version of the Parenting Sense of Competence Scale (C-PSOC) (33). The 6-point rating scale comprises a 17-item and assesses satisfaction and efficacy across two dimensions. Scores range from 17 to 102, with higher scores indicating higher levels of parenting self-efficacy. The C-PSOC has demonstrated high internal consistency and reliability (34).

#### Secondary outcomes

Parenting stress will be measured using the Parenting Stress Index-Short Form (PSI-SF) (35). This questionnaire comprises 36 items and three subscales, namely parenting distress, dysfunctional parent-child interaction, and characteristics of children with difficulties. The questionnaire uses a 5-point Likert scale, ranging from 36 to 180, with higher scores indicating higher levels of parenting stress. The PSI-SF has high reliability and validity (36).

Treatment compliance of parents of children with ASD will be assessed using the Treatment Compliance Scale for Children with Autism Spectrum Disorder (37). The scale consists of two subscales, namely medication compliance (optional) and rehabilitation treatment compliance. This scale employs a 5-point Likert scale, ranging from 1 (never) to 5 (always). The total score ranges from 18 to 110, and when medication items were excluded, the score ranges from 18 to 90. With higher scores indicating better compliance. The scale has good validity and reliability (37).

The family’s quality of life will be measured using the Chinese version of the Beach Center Family Quality of Life Scale (BCFQoL) (38). This 25-item, 5-point Likert scale assesses FQoL across five domains from importance and satisfaction ratings from 1 (not at all important/dissatisfied) to 5 (very important/very satisfied). A higher score indicates a greater level of agreement with the importance or satisfaction of the FQoL. The scale has good reliability and validity (39).

The Chinese version of the Autism Behavior Checklist (ABC) (40) is used to assess parents’ perceptions of the severity of children. The ABC includes 57 items with five dimensions, which are sensory, relating, body and object use, language, and social and self-help, with higher scores indicating greater symptom severity. The cut-off scores for ASD screening and diagnosis were 31 and 62, respectively. The scale has good reliability and validity (41).

#### Acceptability

The participants acceptability of intervention will be assessed by (1) adherence rate, (2) adverse events, and (3) participants’ satisfaction will be assessed by brief open-ended question about suggestions on the improvements of intervention.

### Sample size

The sample size calculation was performed using the Power Analysis and Sample Size (PASS) software (NCSS, Kaysville, Utah). Parenting self-efficacy was chosen as the primary outcome for sample size calculation. According to a study using the C-PSOC to evaluate the effects of psychoeducational therapy on parenting self-efficacy in parents of children with ASD, the mean ± standard deviation (*SD*) of average total parenting self-efficacy was reported as 6.99 (0.76) (26). Considering a 15% dropout rate and α = 0.05 (two-sided), PASS indicated that a total of 80 participants (40 participants in each group) would be required to achieve 80% power.


$$n_{1\;}=\;n_{2}\;=\;2\times [\frac{{\left(t_{\alpha }\;+\;t_{\beta }\right)}^{2}\;\times \;s^{2}}{\delta^{2}}]$$

### Statistical methods

IBM SPSS 26.0 (IBM Corp., NY, USA) will be used for data analysis. Descriptive statistics such as mean, median, standard deviation, interquartile range, frequency, and percentage will be used to describe the baseline characteristics of the participants. The independent *t*-tests and one-way ANOVA will be used to compare the homogeneity of the two groups at baseline. Outcome analyses will be conducted based on the intention-to-treat (ITT) principle. Missing data will be imputed based on the results of Little’s missing completely at random (MCAR) test. The generalized estimating equations (GEE) model will be used to analyze the group and time differences in outcome variables. Potential covariates such as sociodemographic or clinical characteristics with a *P<*0.01 in the baseline will be considered in the adjusted GEE model. Open-ended contents will be summarized descriptively with selected anonymized quotations. Statistical significance will be set at 0.05 (two-sided).

### Monitoring

Before intervention implementation, the researcher used 2 days to train nurses and therapists on all aspects of parent recruitment, data collection, and the treatment protocol. During the intervention process, regular monthly discussions will be conducted within the intervention team to facilitate effective communication and propose resolution of any issues encountered during the intervention.

## Discussion

This study protocol describes the theoretical basis and design of a quasi-experimental study that evaluated the effects of a health education program targeting parental self-efficacy for children with ASD. Early intervention is important for children with ASD, and if the intervention proves to be effective, it will provide important insight into promoting parents’ treatment compliance in early intervention. Improving parental self-efficacy is crucial to improving treatment compliance, FQoL, and reducing parenting stress in parents of children with ASD. Although developed and tested in a Chinese tertiary center, the barriers that lack of parental confidence, skills for home intervention are widely reported across different health system (2). Our results will be applicable for generalization to countries which has limited ASD-related resources.

### Ethics and dissemination

This study is not expected to cause any physical harm for both caregivers and children given the nature of health education. Participants may experience transient fatigue during the program. Researchers are trained to assess participants’ condition and response to fatigue. We will pause education or arrange referral if needed. Adverse events will be recorded and reviewed by the PI within 72 hours. The study protocol will follow the ethical principles outlined by the Helsinki Declaration. Ethics approval has been obtained from the Medical Ethics Committee of the Third Affiliated Hospital of Sun Yat-sen University (No. [2022]02-270-01) prior to the commencement of the study. The eligible parents will be provided with comprehensive information about the purpose, significance, and process of the study before the intervention. Parents will sign informed consent forms and have the right to withdraw from the study at any time. Their decision to withdraw will not affect their subsequent treatment. Additionally, the control group will receive a book valued at 50 CNY at the end of the study. The study protocol will also be available to the parents in the control group.

### Limitations

This study has several limitations. First, this study uses a non-randomized design due to high contamination risk and operational constraints in a single ward. This may increase bias to unbalanced baseline characteristics and susceptibility to secular trends. We will mitigate this via standardized delivery and covariate-adjusted GEE models. Second, the study was conducted at a single center, which may limit the generalizability of the findings to a broader population of parents of children diagnosed with ASD. Third, blinding the intervention practitioners and participants is not feasible due to the nature of the health education program. To mitigate potential bias, data analysts will be blinded. Fourth, this study has not conducted in-depth semi-structured interviews, future studies need to incorporate a planned qualitative interview to deepen understanding of intervention effects.

### Practice implications

The PSE program has the potential to effectively improve parental self-efficacy, treatment compliance, FQoL, and reduce parenting stress and symptom severity in parents and children, respectively. This study can assist pediatric nurses in enhancing early intervention for parents of children with ASD, as they can serve as a manager in multidisciplinary cooperation and resource integration. The study also provides evidence supporting the use of both face-to-face and online sessions in family interventions for parents of children with ASD. Future studies could explore differences in the effects of implementing interventions across different demographic and cultural contexts, providing insights into the development of interventions for parents of children with ASD.

## Conclusion

If the expected results are obtained, the findings of this study will contribute to the development of methods and strategies to improve parental self-efficacy for parents of children with ASD in the early stage of diagnosis. This will be valuable for nurses and healthcare professionals working with this population.

## Authors' note

Protocol version: 3.0, August 2025.
